# Bilateral Video‐Assisted Thoracoscopic Resection for a Patient With Horseshoe Intralobar Pulmonary Sequestration: A Novel Minimally Invasive Approach

**DOI:** 10.1111/ases.70115

**Published:** 2025-07-07

**Authors:** Hiroki Watanabe, Shota Nakamura, Yoshito Imamura, Shoji Okado, Yuji Nomata, Hirofumi Takenaka, Yuta Kawasumi, Keita Nakanishi, Yuka Kadomatsu, Harushi Ueno, Taketo Kato, Tetsuya Mizuno, Masato Mutsuga, Toyofumi Fengshi Chen‐Yoshikawa

**Affiliations:** ^1^ Department of Thoracic Surgery Nagoya University Graduate School of Medicine Nagoya Japan; ^2^ Department of Cardiac Surgery Nagoya University Graduate School of Medicine Nagoya Japan

**Keywords:** horseshoe lung, pulmonary sequestration, video‐assisted thoracic surgery

## Abstract

We encountered a rare case of bilateral intralobar pulmonary sequestration presenting as horseshoe lung. A 44‐year‐old asymptomatic woman was referred to our hospital because of increased serum cancer antigen 125 and carbohydrate antigen 19‐9, accompanied by an abnormal chest shadow. Preoperative imaging indicated a horseshoe‐shaped bilateral intralobar pulmonary sequestration diagnosis. The surgery was initiated on the left side in a semiprone right lateral decubitus position. The aberrant vessels arising from the descending aorta were divided, and after indocyanine green administration, the boundary between the normal and sequestrated lungs was determined and stapled. The left and right pleural cavities were connected, and the left sequestrated lung was pushed into the right thoracic cavity. The right sequestrated lung was similarly resected via the right‐sided approach, and both were resected *en bloc*. Both tumor markers were normalized 3 months postoperatively. We safely performed thoracoscopic surgery by formulating an appropriate surgical plan.

## 1 Introduction

Pulmonary sequestration (PS) is a congenital malformation characterized by the presence of nonfunctional lung tissue that lacks normal bronchial connection and receives its blood supply from an aberrant systemic artery. Based on its pleural covering, PS can be categorized into intralobar PS (IPS) and extralobar PS (EPS). IPS consists of lung parenchyma masses that are contiguous with the adjacent normal lung [1]. Most cases of PS are unilateral, with bilateral ones being very rare. Horseshoe lung is a rare congenital malformation in which the left and right lungs are fused on the dorsal side of the heart. Here, we report a case of bilateral IPS presenting with horseshoe lung that was identified as a result of elevated tumor markers and safely resected via video‐assisted thoracoscopic surgery (VATS). Although managing aberrant arteries branching from the descending aorta and resecting fused bilateral lungs *en bloc* are technically challenging—especially given the need for bilateral surgery—thoracoscopic surgery is a less invasive approach and can significantly reduce the surgical burden on the patient.

## 2 Case Presentation

**FIGURE 1:**
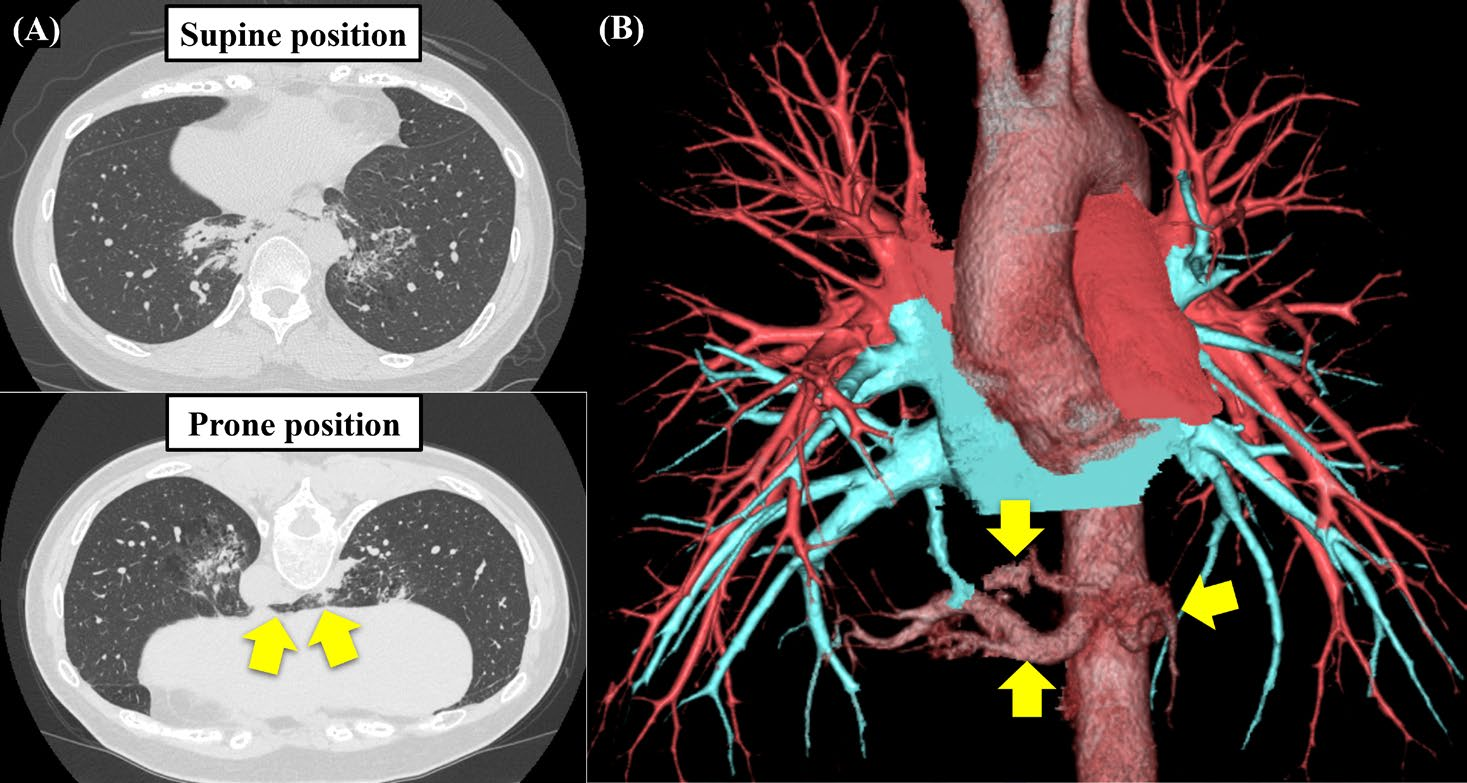
Preoperative chest computed tomography findings. (A) Imaging in the prone position visualizing the horseshoe lung, in which the left and right lungs were fused at the isthmus (arrows). (B) Three aberrant arteries originate from the descending aorta (arrows), whereas the pulmonary veins drain into the normal inferior pulmonary veins on both sides. The computed tomography images were reconstructed in three‐dimensional form with the Ziostation software (Ziosoft Inc., Tokyo, Japan).

**FIGURE 2:**
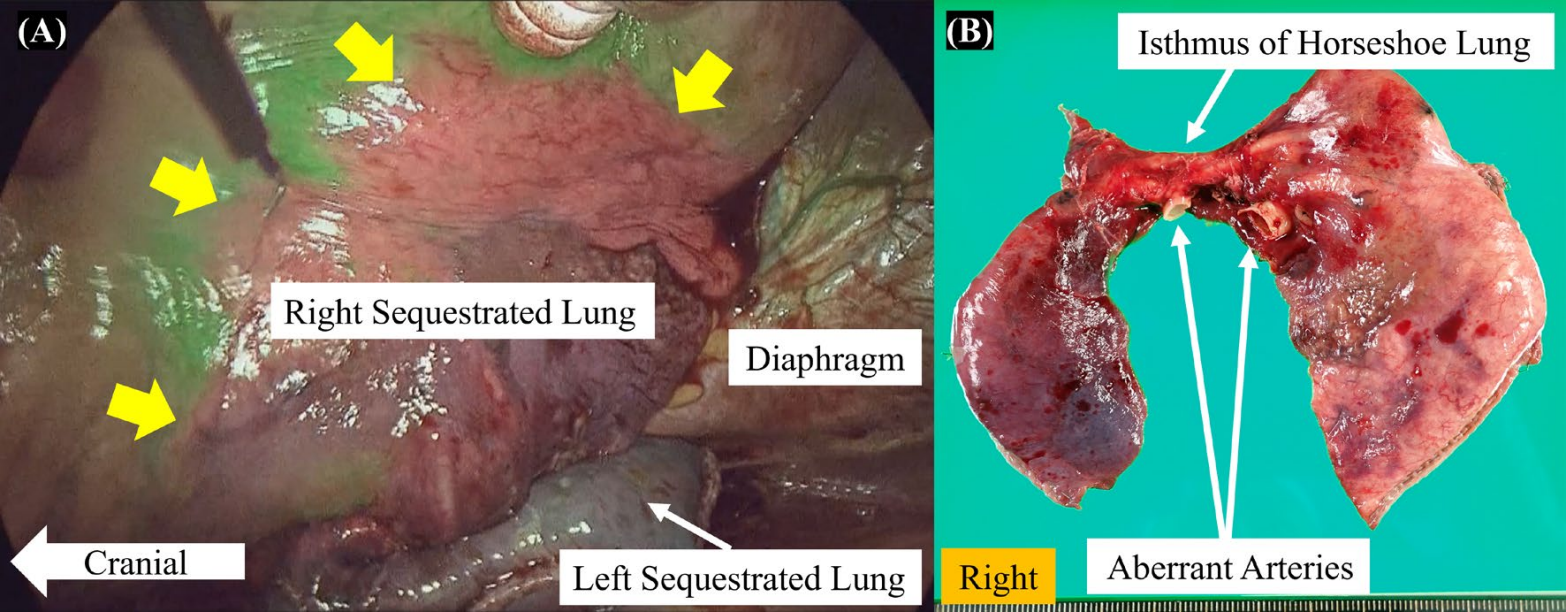
Intraoperative findings. (A) Boundary between the sequestrated lung and the normal lung clearly visualized through indocyanine green administration (arrows). (B) The horseshoe lung was resected *en bloc*. The pathological results revealed that the aberrant vessels demonstrate the structure of elastic arteries, and the lung exhibits severe inflammatory changes, with cystically dilated bronchi containing mucus retention. The findings were consistent with intralobar pulmonary sequestration.

A 44‐year‐old asymptomatic woman was referred to our hospital because of increased serum cancer antigen (CA) 125 (146.0 U/mL) and carbohydrate antigen (CA) 19‐9 (106.0 U/mL) detected during routine health screening, accompanied by an abnormal shadow on chest computed tomography (CT). Chest CT scan revealed reticular and infiltrative shadows in the lower lobes of the bilateral lung as well as a contiguous shadow of the bilateral lungs in the prone position, resulting in the diagnosis of horseshoe lung (Figure 1A). The bronchi in this abnormal area exhibited no connection to the central airways. Three aberrant arteries originated from the descending aorta in the abnormal lungs, whereas the pulmonary veins drained into the normal inferior pulmonary veins on both sides. These findings led to the diagnosis of a horseshoe‐shaped IPS (Figure 1B). Surgery was performed approximately 3 months after referral to our hospital based on the patient's preference for definitive surgical treatment. PS resection along with aberrant blood vessel division was performed via a bilateral three‐port thoracoscopic approach (Supplemental Video S1). The surgery was initiated from the left side, and the patient was placed in a semiprone right lateral decubitus position. The observation hole and surgeon's left‐hand port were inserted in the sixth intercostal space along the left midaxillary line. The surgeon's right‐hand port was inserted in the eighth intercostal space along the left midaxillary line. After dissecting and dividing the three aberrant arteries from the aorta, an anesthesiologist administered indocyanine green (ICG) intravenously at a dose of 7.5 mg/body. The boundary between the normal and sequestrated lungs was determined. The left sequestrated lung was stapled along the boundary, including division of the outflow pulmonary vein. The left and right thoracic cavities were connected by adequately dissecting the space between the descending aorta and esophagus. The resected left sequestrated lung was pushed into the right thoracic cavity, completing the left‐sided procedure. This maneuver is essential for *en bloc* resection of the horseshoe lung, in which the right and left lungs are fused. The most important step in this procedure is mobilizing the left sequestrated lung, isthmus, and part of the right sequestrated lung from the left thoracic cavity. The patient was then positioned similarly to the left side, and the right‐sided procedure was initiated. Using a three‐port VATS approach, as performed on the contralateral side, the right sequestrated lung was resected after identifying the boundary between the right sequestrated lung and normal lung following ICG administration (Figure 2A), and the horseshoe lung was resected *en bloc* (Figure 2B). The operative time was 281 min, and the blood loss was 80 g. The postoperative course was uneventful, and the patient was discharged 10 days postoperatively. These results were consistent with those of IPS that pathologically presents as a horseshoe lung. Both serum CA125 and CA19‐9 levels were normalized 3 months postoperatively, with CA125 decreasing to 15.5 U/mL and CA19‐9 to 9.0 U/mL. At 1‐year follow‐up, the patient remained asymptomatic and in good general condition.

## 3 Discussion

Here, we report a case of IPS presenting as a horseshoe lung with a bilateral connection in the posterior mediastinum that was successfully resected through thoracoscopic surgery. To the best of our knowledge, this is the first reported case of IPS in which resection was performed using a minimally invasive VATS approach. The preoperatively increased CA125 and CA19‐9 levels normalized immediately after PS resection, indicating a successful abnormal lung tissue removal.

Horseshoe lung is a rare congenital malformation in which the isthmus of the pulmonary parenchyma extends from the right lung base across the midline behind the pericardium and fuses with the left lung base [2]. Horseshoe lung and mediastinal lung herniation involve the lower lobe that extends to the opposite side [3]. Figa et al. classified horseshoe lungs based on pleural structure. They revealed that the pleural cavities remain distinct if four pleural layers separate the crossover lung tissue from the contralateral lung, indicating mediastinal herniation rather than horseshoe lung. This was not the case in our patient. Six cases of surgically treated horseshoe lung in adults have been reported; of these, three were treated via open thoracotomy [4, 5], two via VATS, and one via robot‐assisted thoracoscopic surgery (RATS). One of the three cases treated via open thoracotomy developed postoperative pneumonia and underwent open‐window surgery for concomitant pyothorax [6]. Meanwhile, both cases treated using VATS involved the transection of the horseshoe lung at the isthmus. Furthermore, the contralateral horseshoe lung was resected in one case, whereas it was left intact in the other case [7]. One case that was treated with RATS had IPS and EPS on the right and left sides, respectively, and the surgery was performed through a bilateral approach [8]. To the best of our knowledge, our case is the first in which complete endoscopic resection of bilateral IPS was performed.

In this case, we employed four strategies to perform endoscopic surgery. First, the working space was secured by placing the patient in a semiprone lateral decubitus position to shift the lung ventrally, enabling working space establishment and posterior mediastinum visualization, further facilitated by maintaining artificial pneumothorax. This allowed for safer manipulation, including aberrant vessel management, and reduced the number of assist ports by one. Second, by approaching from the left side first, we managed the aberrant vessels—the most crucial and hazardous step of this surgery—at the very beginning, as this approach facilitated clear visualization of the descending aorta. Third, using the isthmus of the horseshoe lung and adequately dissecting the esophagus and descending aorta, we connected the bilateral pleural cavities and pushed the left sequestrated lung into the right thoracic cavity. Fourth, we visualized the lung boundaries to be resected and accurately divided the sequestrated lung by intravenously administering ICG. This was an IPS case; thus, the boundary between the normal lung and the sequestrated lung was ambiguous. We have previously reported ICG administration in PS [9, 10]. The sequestrated lung has a different blood supply from the normal lung; thus, after dividing the vessels supplying blood to the sequestrated lung, ICG was administered to clearly visualize the boundary between the normal lung and the sequestrated lung.

CA19‐9 and CA125 have been reported to be produced by normal bronchial epithelial cells. In our case, pathological findings suggested inflammation and mucus retention within the sequestrated lung, which was presumed to be the cause of tumor marker elevation.

In conclusion, we safely performed thoracoscopic resection of the sequestrated lung presenting as a horseshoe lung by establishing an appropriate surgical plan.

## Author Contributions

H.W. conceived the primary hypothesis, designed the research, collected the data, and wrote the manuscript. S.N. conceived the primary hypothesis, designed the research, and edited the manuscript. Y.I., S.O., Y.N., H.T., Y.K., K.N., Y.K., H.U., T.K., T.M., and M.M. edited the manuscript. T.F.C.‐Y. supervised the research and edited the manuscript. All of the authors have read and approved the final version of the manuscript.

## Ethics Statement

The institutional review board of Nagoya University Hospital approved the study protocol (No. 2024‐0326). The patient provided informed consent.

## Conflicts of Interest

The authors declare no conflicts of interest.

## Supporting information


**Video S1.** The surgery was initiated from the left side, and the patient was placed in a semiprone right lateral decubitus position to shift the lung ventrally and improve dorsal organ visualization. The GelPOINT Mini Advanced Access Platform (Applied Medical, Rancho Santa Margarita, CA) was inserted in the sixth intercostal space of the left midaxillary line for the observation hole and the surgeon’s left‐hand port. The AirSeal Access Port (CONMED, Utica, NY) was inserted in the eighth intercostal space of the left midaxillary line for the surgeon’s right‐hand port. After dissecting the three aberrant arteries that branch from the aorta, they were clipped, stapled, and divided. Further, the inflowing pulmonary vein was stapled and divided. After dissecting the aberrant arteries, an anesthesiologist administered indocyanine green. The boundary between the normal and sequestrated lungs was determined using the real‐time IMAGE 1 S D‐LIGHT P SCB, RUBINA (Karl Storz, Germany). The left sequestrated lung was stapled along the boundary, including division of the outflow pulmonary vein. The left and right thoracic cavities were connected by adequately dissecting the space between the descending aorta and the esophagus. The resected left sequestrated lung was pushed into the right thoracic cavity, completing the left‐sided procedure. The patient was then placed in a semiprone left lateral decubitus position, similar to the opposite side, and the right‐sided procedure was initiated. Using a three‐port video‐assisted thoracoscopic surgery with artificial pneumothorax approach, as performed on the contralateral side, the right sequestrated lung was resected after identifying the boundary between the right sequestrated lung and the normal lung with indocyanine green administration, and the horseshoe lung was resected *en bloc*.

## Data Availability

The data that support the findings of this study are available from the corresponding author upon reasonable request.
